# Immunoproteasome-Deficiency Has No Effects on NK Cell Education, but Confers Lymphocytes into Targets for NK Cells in Infected Wild-Type Mice

**DOI:** 10.1371/journal.pone.0023769

**Published:** 2011-08-24

**Authors:** Mary J. G. van Helden, Natascha de Graaf, Cornelis P. J. Bekker, Claire J. P. Boog, Dietmar M. W. Zaiss, Alice J. A. M. Sijts

**Affiliations:** 1 Division of Immunology, Faculty of Veterinary Medicine, University of Utrecht, Utrecht, The Netherlands; 2 Department of Vaccinology, Centre for Infectious Disease Control, National Institute for Public Health and the Environment (RIVM), Bilthoven, The Netherlands; Centre de Recherche Public de la Santé (CRP-Santé), Luxembourg

## Abstract

Natural killer (NK) cells are part of the innate immune system and contribute to the eradication of virus infected cells and tumors. NK cells express inhibitory and activating receptors and their decision to kill a target cell is based on the balance of signals received through these receptors. MHC class I molecules are recognized by inhibitory receptors, and their presence during NK cell education influences the responsiveness of peripheral NK cells. We here demonstrate that mice with reduced MHC class I cell surface expression, due to deficiency of immunoproteasomes, have responsive NK cells in the periphery, indicating that the lower MHC class I levels do not alter NK cell education. Following adoptive transfer into wild-type (*wt*) recipients, immunoproteasome-deficient splenocytes are tolerated in naive but rejected in virus-infected recipients, in an NK cell dependent fashion. These results indicate that the relatively low MHC class I levels are sufficient to protect these cells from rejection by *wt* NK cells, but that this tolerance is broken in infection, inducing an NK cell-dependent rejection of immunoproteasome-deficient cells.

## Introduction

NK cells are part of the innate immune system and play a role in the eradication of viruses and tumors. NK cells express a variety of germ-line encoded inhibitory and activating receptors that interact with their respective ligands on target cells [1]. Upon interaction with a potential target cell, the NK cell's decision whether to attack this cell is determined by the sum of signals received through these receptors [2]. MHC class I molecules are recognized by inhibitory receptors, and therefore, NK cells attack cells that lack cell surface MHC class I [3]. This ‘missing self’ hypothesis would imply that NK cells from mice that lack MHC class I expression, like β_2_m- or TAP-deficient mice, reject their own cells. This is, however, not the case because developing NK cells are educated in a MHC class I-dependent process called ‘licensing’ [4] or ‘disarming’ [5], resulting in responsive NK cells in the periphery. NK cells from mice that lack MHC class I expression are therefore hyporesponsive [6], [7]. The recognition of MHC class I by murine NK cells is largely dependent on inhibitory receptors of the Ly49 family [8]. The responsiveness of peripheral NK cells is thus determined by presence of MHC class I on the target cell, and by expression of Ly49 on the NK cell. This responsiveness is tunable like a rheostat, thus, changes in strength of inhibitory signals during education quantitatively tune NK cell activity [9]–[11]. Consequently, mice that express different types and combinations of MHC class I alleles have functionally different NK cells as more inhibitory signals during education result in increasingly responsive NK cells [9], [12]. Joncker *et al*. showed that NK cell responsiveness correlated with the number of inhibitory self-MHC class I receptors on NK cells [11].

Previous studies have shown that lymphoid cells of β2m-, TAP- or tapasin-deficient mice, which fail to express or display low amounts of MHC class I molecules on their cell surface, are rejected by wild-type (*wt*) NK cells [13]–[15]. MHC class I cell surface trafficking requires the presence of peptide in the antigen processing groove. These peptides usually are generated by or following proteasome-mediated proteolysis. The ability of proteasomes to degrade proteins and generate high affinity MHC class I-binding peptides is determined by the identity of the proteasome' catalytic subunits. In most tissues, proteasome-mediated proteolysis is exerted by three constitutively expressed proteasome subunits. In contrast, cells exposed to (pro) inflammatory cytokines as well as lymphoid cells express three inducible proteasome subunits, named β1i/LMP2, β2i/MECL-1 and β5i/LMP7 that incorporate into so called immunoproteasomes and enhance the proteasome-mediated generation of high affinity MHC class I binding peptides. Conversely, deficiency of these subunits reduces the abundance of MHC class I molecules on the cell surface [16]–[19]. Since the responsiveness of peripheral NK cells is tuned by strength of inhibitory signals experienced during education, we here determined whether the relatively low quantities of cell surface MHC class I molecules in β2i/MECL-1& β5i/LMP7-deficient mice modify the responsiveness of peripheral NK cell in these mice. In addition, as different studies already showed that lymphoid cells lacking one or more immunoproteasome subunits are rejected upon transfer in infected *wt* mice [20]–[22], we determined whether immunosubunit deficiency render lymphoid cells into targets for *wt* NK cells in infected *wt* recipient mice. Our data indicate that peripheral NK cells in immunoproteasome-deficient mice are normally responsive. Transferred immunoproteasome-deficient splenocytes were tolerated in *wt* recipients, but rejected in an NK cell-mediated fashion following infection of the recipient mice.

## Results

### Reduced MHC class I cell surface expression on DCs of immunosubunit- & RAG1-deficient mice

**Figure 1:**
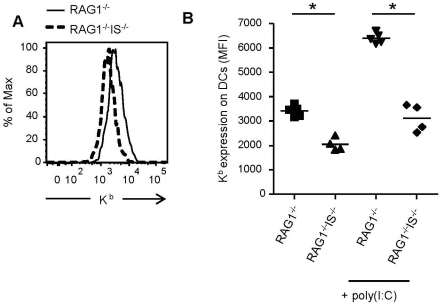
Effect of immunoproteasome-deficiency on constitutive expression and activation-induced upregulation of MHC class I molecules. Splenocytes of untreated or poly(I:C) treated RAG1^−/−^ and RAG1^−/−^β2i/MECL-1^−/−^β5i/LMP7^−/−^ (RAG1^−/−^IS^−/−^) mice were analyzed for the expression of H2-K^b^ on DCs (CD11c^hi^CD11b^int^). (A) Representative histogram of untreated mice showing H2-K^b^ expression on DCs. (B) H2-K^b^ mean fluorescent intensity (MFI) on DCs of individual mice. Data are representative of three independent experiments, n = 4–6 mice per group. Statistical analysis was performed using a Mann-Whitney *U* test. *, P<0.05.

During development, NK cell education depends on interactions between MHC class I molecules and NK cell-expressed inhibitory receptors. It has been shown previously that mice deficient for immunoproteasomes express reduced levels of cell-surface MHC class I molecules [16], [18], [19]. To investigate whether immunoproteasomes, by reducing MHC class I expression, alter NK cell education, we used β5i/LMP7- plus β2i/MECL-1-deficient mice bred onto a RAG1-deficient background (RAG1^−/−^), which have relatively high numbers of NK cells. Consistent with previous data in immunocompetent *wt* and β2i/MECL-1& β5i/LMP7-deficient mice, amounts of MHC class I H2-K^b^ molecules expressed on splenic dendritic cells (DCs) and other lymphoid cells of RAG1^−/−^ β2i/MECL-1^−/−^ β5i/LMP7^−/−^ mice were considerably lower than those on DCs of RAG1-deficient controls (Figure 1A, B and data not shown). Infection of mice with *Listeria monocytogenes* upregulates MHC class I expression in infected tissue [18], [19]. In line with these findings, treatment of RAG1^−/−^ mice with the immunostimulator poly(I:C) led to upregulation of H2-K^b^ cell surface expression on DCs of these mice (Figure 1B and data not shown). Also DCs in poly(I:C)-treated β2i/MECL-1& β5i/LMP7&RAG1-deficient mice upregulated MHC class I H-2K^b^ expression, but to a lesser extent than DCs in RAG1-deficient mice. The reduced ability of immunosubunit-deficient DCs to upregulate MHC class I cell surface expression reflects a defect in the supply of high affinity peptides available for binding to MHC class I molecules [19].

### Responsiveness of peripheral NK cells in immunosubunit-deficient mice

**Figure 2:**
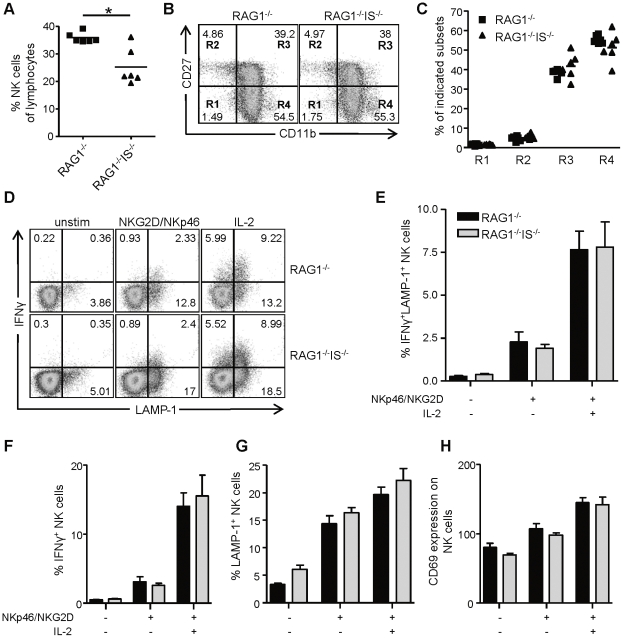
Immunoproteasome-deficient mice have responsive peripheral NK cells. (A–C) Splenocytes of RAG1^−/−^ and RAG1^−/−^β2i/MECL-1^−/−^β5i/LMP7^−/−^ (RAG1^−/−^IS^−/−^) mice were analyzed by flow cytometry (A) Frequencies of NK cells (DX5^+^NKp46^+^) as percentage of total lymphocytes. (B,C) Expression of CD11b and CD27 on DX5^+^NKp46^+^ NK cells. (B) Representative FACS plots and (C) graph showing percentages of subsets for individual mice for the regions indicated in (B). (D–H) Splenocytes of RAG1^−/−^ and RAG1^−/−^IS^−/−^ mice were incubated on plates coated with anti-NKG2D and anti-NKp46, in the presence or absence of IL-2, or left unstimulated. Frequencies of IFNγ^+^, LAMP-1^+^ NK cells and CD69 mean fluorescent intensity (MFI) on NK cells were determined by flow cytometry. (D) Representative FACS plots showing IFNγ^+^ and LAMP-1^+^ NK cells. (E–H) Bars showing the percentages of IFNγ^+^LAMP-1^+^ NK cells (E), IFNγ^+^ NK cells (F), LAMP-1^+^ NK cells (G), and CD69 MFIs on NK cells (H). Results are shown as mean ± S.E.M. Data are representative for 2 independent experiments with 6 mice per group. Statistical analysis was performed using Mann-Whitney U test. *, P<0.05.

The responsiveness of peripheral NK cells is quantitatively tuned by the number of MHC class I molecules that are present during NK cell development [9]–[11]. We therefore assessed the responsiveness of peripheral NK cells in immunosunit-deficient mice, which have reduced MHC class I levels. We first measured some general aspects and consistently found that the numbers of NK cells in the spleens of RAG1^−/−^ β2i/MECL-1^−/−^ β5i/LMP7^−/−^ mice were significantly lower than in RAG1^−/−^ mice (Figure 2A). We furthermore analyzed CD27 and CD11b expression on NK cells from RAG1^−/−^ β2i/MECL-1^−/−^ β5i/LMP7^−/−^ and RAG1^−/−^ mice to define their maturation status. The most immature NK cells are CD11b^−^CD27^−^ (R1) and during maturation they become CD11b^−^CD27^+^ (R2), CD11b^+^CD27^+^ (R3) and finally CD11b^+^CD27^−^ (R4), respectively [23], [24]. Comparing the two groups of mice, we found that the expression of these markers were highly comparable (Figure 2B, C). To assess the responsiveness of NK cells, we stimulated splenocytes from RAG1^−/−^ and RAG1^−/−^ β2i/MECL-1^−/−^ β5i/LMP7^−/−^ mice with plate-bound antibodies against NKp46 and NKG2D and measured CD69 upregulation, the production of IFNγ, and degranulation using LAMP-1. Levels of CD69, IFNγ, and LAMP-1 were increased on antibody-stimulated NK cells and there were no significant differences between the two groups of mice (Figure 2D–H). Furthermore, when IL-2 was added during incubation, NK cells from RAG1^−/−^ and RAG1^−/−^ β2i/MECL-1^−/−^ β5i/LMP7^−/−^ mice upregulate CD69, IFNγ, and LAMP-1 in a similar fashion (Figure 2D–H). Together, these results show that splenic NK cells from RAG1^−/−^ β2i/MECL-1^−/−^ β5i/LMP7^−/−^ and RAG1^−/−^ mice are similarly responsive.

### NK cell-dependent rejection of immunosubunit-deficient cells in infected recipients

**Figure 3:**
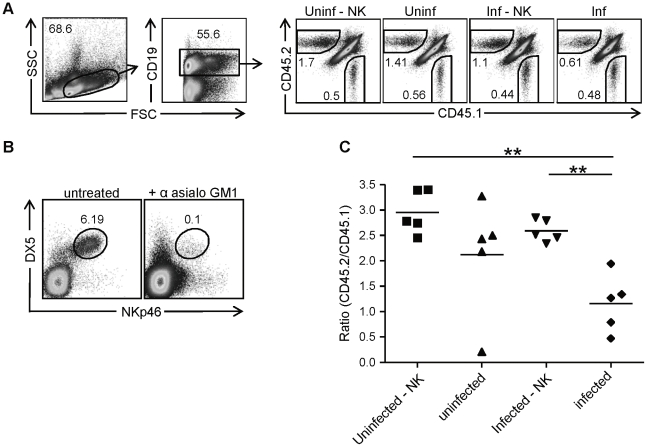
Rejection of immunosubunit-deficient cells in influenza virus infected mice. Splenocytes of congenic CD45.1 (*wt*) and CD45.2 β2i/MECL-1^−/−^β5i/LMP7^−/−^ (IS^−/−^) mice were 1∶1 mixed based on total cell numbers, and transferred *i.v.* into untreated or anti-asialo GM1 treated CD45.1.2 recipients, that were subsequently infected *i.n*. with influenza virus or left uninfected. Recipient mice were sacrificed 8 days later and spleens were analyzed. (A) Representative FACS plots showing gating strategies and percentages of recovered CD45.1^+^ (*wt*) and CD45.2^+^ (IS^−/−^) cells of CD19^+^ cells in the different groups: uninf – NK (uninfected, anti-asialo GM1 treated), uninf (uninfected, untreated), inf – NK (influenza virus infected, anti-asialo GM1 treated), inf (influenza virus infected, untreated). (B) Representative FACS plots gated on TCRβ^−^ cells showing staining for DX5 and NKp46 on splenocytes from untreated and anti-asialo GM1 treated mice. Cells in the gate are DX5^+^NKp46^+^ NK cells. (C) Ratio of CD45.2 (IS^−/−^)/CD45.1 (*wt*) calculated by dividing absolute numbers of CD19^+^ CD45.2^+^ (IS^−/−^) cells by absolute numbers of CD19^+^ CD45.1^+^ (*wt*) cells. Results are representative for 2 independent experiments with 5–6 mice per group. Statistical analysis was performed using a Mann-Whitney *U* test. **, P<0.01.

Several groups have previously reported that immunosubunit-deficient cells are rejected after transfer into infected *wt* recipients [20]–[22]. Reasoning that the relatively low cell surface levels of MHC class I molecules on β2i/MECL-1^−/−^ β5i/LMP7^−/−^ cells might reduce the strength of inhibitory signals received by NK cells in infected *wt* recipients, we decided to investigate whether NK cells contributed to the observed rejection. Splenocytes of congenic *wt* CD45.1^+^ and CD45.2^+^ β2i/MECL-1^−/−^ β5i/LMP7^−/−^ mice were mixed and transferred *i.v*. into *wt* CD45.1.2 recipients that either had been treated with anti-asialo GM1 to deplete NK cells (Figure 3B) or had been left untreated. Subsequently, the recipients were infected *i.n*. with influenza virus or left uninfected. Eight days later, mice were sacrificed and percentages of donor-derived B cells were determined by FACS analysis (Figure 3A). In uninfected recipient mice, the numbers of recovered immunosubunit-deficient CD45.2 B cells exceeded those of CD45.1 cells (Figure 3C), which reflected the relatively high frequencies of B cells in β2i/MECL-1^−/−^ β5i/LMP7^−/−^ mouse spleens. No significant differences in relative quantities of β2i/MECL-1^−/−^ β5i/LMP7^−/−^ B cells were detected between NK cell depleted and undepleted naïve mice (Figure 3C) indicating that β2i/MECL-1^−/−^ β5i/LMP7^−/−^ B cells were tolerated by NK cells in naïve *wt* recipients. Conversely, after infection with influenza virus, the relative proportions of recovered β2i/MECL-1^−/−^ β5i/LMP7^−/−^ cells were dramatically decreased in undepleted compared to NK cell depleted mice (Figure 3C). Thus, although tolerated by naïve recipients, a relatively high proportion of β2i/MECL-1^−/−^ β5i/LMP7^−/−^ cells is rejected by NK cells in infected *wt* recipient mice.

### Splenocytes from poly(I:C)-treated immunoproteasome-deficient mice are tolerated by *wt* NK cells in naïve mice

**Figure 4:**
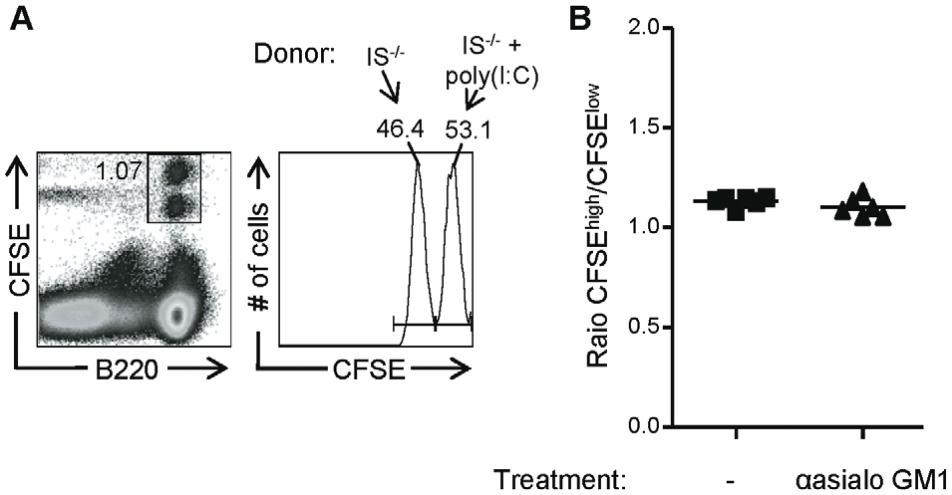
Adoptively transferred splenocytes of poly(I:C)-treated immunoproteasome-deficient mice are tolerated by NK cells in naïve wt recipients. CFSE-labeled splenocytes of untreated β2i/MECL-1−/−β5i/LMP7−/− (IS−/−; CFSElow) and of poly(I:C) treated IS−/− (CFSEhigh) mice were mixed 1∶1 and transferred i.v. into untreated or anti-asialo GM1 treated wt recipients. Recipient mice were sacrificed 17 h later and spleens were analyzed. (A) Representative FACS plots showing gating strategies. (B) Ratios of poly(I:C) treated (CFSEhigh) and untreated (CFSElow) IS−/− cells calculated by dividing absolute numbers of B220+CFSEhigh cells by absolute numbers of B220+CFSElow cells. Results represent one experiment with 6 mice per group.

Our data thus far showed that immunoproteasome-deficient splenocytes were rejected by *wt* NK cells in infected recipients. This increased sensitivity to NK cell-mediated lysis might result from inflammation-induced changes of the target cells, from changes in NK cell sensitivity (i.e. priming; [25]), or from a combination of the two. To address these possibilities, a mix of CFSE-labeled splenocytes of untreated β2i/MECL-1& β5i/LMP7-deficient (CFSE^low^) and poly(I:C) treated β2i/MECL-1& β5i/LMP7-deficient (CFSE^high^) mice was transferred into naïve untreated and anti-asialo GM1-treated *wt* recipients. Adoptively transferred β2i/MECL-1& β5i/LMP7-deficient splenocytes of naïve mice are tolerated by NK cells in naïve *wt* mice (Figure 3) and thus served as internal control. Approximately 17 h after transfer, recipient mice were sacrificed and the relative proportions of donor-derived splenocytes were determined by FACS analysis. Figure 4 shows that the ratios between β2i/MECL-1& β5i/LMP7-deficient cells of poly(I:C)-treated and untreated mice were similar both in untreated and NK cell-depleted recipient mice. These data indicate that adoptively transferred β2i/MECL-1&β5i/LMP7-deficient cells of poly(I:C)-treated mice are tolerated by NK cells in naïve *wt* recipients.

## Discussion

Previous studies have shown that defects in MHC class I antigen processing that reduce MHC class I cell surface expression, such as caused by for example a lack of β_2_m or tapasin, are accompanied by hyporesponsiveness of peripheral NK cells [6], [7]. In the current study, we show that immunoproteasome-deficient mice, despite diminished MHC class I cell surface expression, have responsive peripheral NK cells. We furthermore show that adoptively transferred immunoproteasome-deficient splenocytes are tolerated by *wt* NK cells in naïve mice, however, are rejected by NK cells in influenza virus infected mice. These data suggest that decreased MHC class I surface expression due to a lack of immunoproteasome expression, enhances the susceptibility of cells to NK cell mediated lysis in infected recipients.

We decided to assess the responsiveness of NK cells of immunoproteasome-deficient mice, since NK cells are educated in a MHC class I-dependent fashion, and the level of MHC class I influences the NK cell responsiveness [9]–[11]. Although immunoproteasome-deficient mice have lower MHC class I levels (Figure 1), they have normal responsive NK cells in the periphery (Figure 2 D–H). These results seem in contrast with the rheostat model, which states that the responsiveness of peripheral NK cell is tuned by the amount of inhibitory signals received during education. Höglund's group studied the quantitative aspect of NK cell education by using mice that expressed different MHC class I alleles [9], [10] and found a relation between the inhibitory input during education and NK cell function in the periphery. Joncker *et al*. showed that the number of receptors expressed for self-MHC class I on NK cells correlated with their responsiveness [11]. However, in a recent paper, Jonsson *et al*. studied education of Ly49A^+^ NK cells quantitatively by using a group of MHC congenic mice [26]. These mice expressed different MHC class I alleles on the same genetic background. By using Ly49A tetramers, they measured the affinity of these different MHC class I alleles for Ly49A, and compared this to educational capacity. Although their results were largely concordant to the rheostat model, these authors showed that optimal NK cell education already occurred with moderate-binding MHC class I ligands. Furthermore, H2-D^d^ and H2-D^k^ hemizygous mice, that showed a reduction in MHC class I levels of roughly 50%, had educated NK cells in the periphery, supporting a low saturation threshold for NK cell education. These findings are in agreement with our results in RAG1^−/−^β2i/MECL-1^−/−^β5i/LMP7^−/−^ mice, where the decrease in MHC class I cell surface levels is approximately 50%, in the absence of immune stimulation (Figure 1).

Since immunoproteasome-deficient mice express lower levels of MHC class I molecules on the cell surface, we also tested whether these cells are targets for NK cell-mediated rejection upon adoptive transfer into *wt* recipients. We found that immunoproteasome-deficient B cells of β2i/MECL-1&β5i/LMP7-deficient C57BL/6 mice, which displayed an approximately 30% reduction in MHC class I levels [19], were tolerated by NK cells in *wt* C57BL/6 mice. This is in line with a report by Brodin *et al*., showing that cells of different types of MHC class I hemizygous mice, which have up to 60% reduced MHC class I levels, are tolerated by *wt* NK cells [27]. In these studies, NK cell-mediated rejection occurred only when MHC class I levels dropped below 80% of their original level.

Recent studies suggested that adoptively transferred immunoproteasome deficient T cells failed to survive in infected recipients [20]–[22]. To test whether NK cells are responsible for this loss, we adoptively transferred immunoproteasome-deficient splenocytes into NK cell depleted and untreated recipients, which were subsequently infected with influenza virus. Although immunoproteasome-deficient cells were tolerated in naïve recipients, our results show a NK cell-dependent rejection in infected recipients (Figure 3C). This finding complements a study by Moebius *et al.*, who tested different mechanisms for T cell loss, and concluded that immunoproteasomes were essential for survival and expansion of T cells in infected mice [22]. In these studies NK cell-mediated rejection was considered as unlikely, as transferred immunoproteasome-deficient CD8 T cells were tolerated by uninfected recipients, and because also LMP2- and MECL-1- deficient T cells were lost, although to a lesser extent than LMP7-deficient cells [22]. From these and our studies, we conclude that the reason for loss of immunosubunit-deficient cells in infected *wt* mice is complex and involves multiple mechanisms, including NK cell mediated rejection and a survival/expansion disadvantage.

Sun and Lanier have shown that β_2_m^−/−^∶*wt* mixed BM chimeric mice lose tolerance upon MCMV infection, resulting in NK cell-mediated rejection of β_2_m^−/−^ cells [28]. This result resembles our finding, although we detected NK cell-dependent rejection of an otherwise tolerated cell-population, while β_2_m^−/−^ cells are rejected by NK cells in a naïve mouse. We postulate that the rejection of immunoproteasome-deficient cells in infected recipients is the net result of two events. First, we showed that upon inflammation, immunoproteasome deficient cells upregulated MHC class I to a lesser extent than *wt* NK cells (Figure 1). Since inflammation also causes upregulation of activating ligands (Raulet et al, 2003), presumably in immunoproteasome-deficient cells as in *wt* cells, an altered balance of activating and inhibitory ligands might account for the NK cell mediated attack of immunoproteasome-deficient cells in infected *wt* recipients. Secondly, infection induces priming in the LN and thereby renders NK cells fully functional [25]. To study the role of NK cell priming, we adoptively transferred splenocytes of poly(I:C) treated immunoproteasome-deficient mice into naïve *wt* mice (Figure 4). Despite a suboptimal, poly(I:C)-induced upregulation of MHC class I molecules (Figure 1B) in face of a presumably normal upregulation of activating ligands, these splenocytes were tolerated by NK cells in recipient mice. We therefore conclude that NK cell priming in the *wt* recipient mice is required to enhance NK cell sensitivity, which then leads to rejection immunoproteasome-deficient cells, recognized due to a disbalance between activating (normal) and inhibitory ligands (reduced).

In conclusion, we here have shown that immunoproteasome-deficient mice, despite a lower abundance of cell surface MHC class I molecules, have normally responsive NK cells in the periphery. Lymphoid cells of immunoproteasome-deficient cells are tolerated by NK cells in naïve *wt* mice, but this tolerance is broken upon infection.

## Materials and Methods

### Mice, infection and poly(I:C) stimulation

C57BL/6 (B6).SJL (CD45.1), CD45.1.2 (F1 of B6×B6.SJL), RAG-1^−/−^ and β5i/LMP7^−/−^β2i/MECL-1^−/−^ mice [17] were maintained by in-house breeding under standard conditions and were used between 7–14 weeks of age. RAG1^−/−^ mice were crossed with β2i/MECL-1&β5i/LMP7 gene-deficient mice to generate β5i/LMP7&β2i/MECL-1&RAG1 gene-deficient mice. PSMB8 (iβ5/LMP7)-deficiency was detected by PCR, using the oligonucleotides 5′- GGACCAGGACTTTACTACGTAGATG-3′ on PSMB8 or 5′-CCGACGGCGAGGATCTCGTCGTGA-3′ on the neomycin cassette as forward and the PSMB8-specific oligonucleotide 5′-CTTGTACAGCAGGTCACTGACATCG-3′ as reverse primer. To detect PSMB10 (iβ2/MECL-1)-deficiency, the oligonucleotides 5′-CAGAGAGAAACACGTGACAGACTGG-3′ on PSMB10 or 5′- CCGACGGCGAGGATCTCGTCGTGA-3′ on the neomycin cassette were used as forward and the PSMB10-specific oligonucleotide 5′- CAGGACAGGTGTGGTTCCAGGAGC-3′ as reverse primer. RAG1-deficiency was verified by flow cytometry, using an anti-TCRβ and anti-CD19 antibody. Infection with influenza virus (A/HK/×31; H3N2) was performed *i.n.* under light isofluorane anesthesia with 10^5^ 50% egg infective dose in 30 µl PBS as described [29]. For stimulation *in vivo*, mice were injected *i.v.* with 100 µg poly(I:C) in PBS 24 h before sacrifice.

### Ethics Statement

All animal experiments were carried out in strict accordance with the Dutch Animal Experimentation Act and EU directives 86/609/CEE and 2010/63/EU related to the protection of vertebrate animals used for experimental or other scientific purposes. The experimental protocols were approved by the Committee on Animal Experiments of the University of Utrecht (DEC 2010.II.12.216 and 2010.II.08.153) and performed in the Central Laboratory Animal Research Facility of the University of Utrecht, which has AAALAC (Association for Assessment and Accreditation of Laboratory Animal Care) accreditation.

### Cell isolation, staining and flow cytometry

Mice were sacrificed by cervical dislocation, spleens were excised, leukocytes were obtained by pressing through a 70-µM cell strainer (BD Biosciences), and red blood cells were removed by ammonium chloride lysis. Staining of surface markers with the indicated antibodies was performed in the presence of Fc block (2.4G2) for at least 20 minutes on ice. For intracellular staining, cells were fixed with 2% paraformaldehyde for 20 minutes at room temperature, and intracellular staining was performed in the presence of 0.5% saponine for 1 hour at 4°C. Antibodies were purchased from eBioscience [anti-CD49b (DX5), -CD27 (LG.7F9), -CD11b (M1/70), -CD11c (N418), -H2-K^b^ (AF6-88.5.5.3), -CD19 (MB19-1), -NKp46 (29A1.4), -CD45.2 (104), -IFNγ (XMG1.2), -CD69 (MB19-1)] or from Biolegend [anti-LAMP-1 (1D4B), -TCRβ (H57-597), -CD45.1 (A20)]. Samples were measured on a FACSCantoII (BD Biosciences) and analyzed with FlowJo software (Tree Star).

### *Ex vivo* stimulation of NK cells

High protein binding EIA/RIA plates (Costar) were left uncoated or were O/N coated with anti-NKG2D (10 µg/ml) and anti-NKp46 (10 µg/ml) in PBS, and approximately 0.5×10^6^ splenocytes of RAG1^−/−^ or of RAG1^−/−^β5i/LMP7^−/−^β2i/MECL-1^−/−^ mice were incubated on these plates in the presence or absence of 10 µg/ml IL-2. For CD69 measurements, cells were incubated for 6 hours, and for LAMP-1 and IFNγ measurements, cells were incubated for 2 hours in the absence of and an additional 6 hours in the presence of 10 µM monensin and 1,25 µg anti-LAMP-1.

### Adoptive transfer

Splenocytes of CD45.1 and β5i/LMP7^−/−^β2i/MECL-1^−/−^ were mixed and approximately 15×10^6^ cells were transferred *i.v.* into CD45.1.2. recipients that were left untreated, NK cell depleted, or influenza virus infected as indicated. NK cells were depleted by *i.p.* (day −1 and day 4) and *i.v.* (at the time of adoptive transfer) administration of 25 µl anti-asialo GM1 antisera (Wako) in 200 µl in PBS. Recipients were sacrificed 8 days later, and the recovery of CD45.1 and CD45.2 cells was measured by flow cytometry as described. To adoptively transfer CFSE-labeled immunoproteasome-deficient cells, splenocytes of untreated and poly(I:C) treated β2i/MECL-1^−/−^β5i/LMP7^−/−^ mice were incubated with 0.4 and 4 µM CFSE (Invitrogen), respectively, for 8 minutes at room temperature. After quenching of CFSE with FCS, the cells were thoroughly washed with PBS, mixed 1∶1 and approximately 10×10^6^ cells were transferred *i.v.* into untreated or NK cell depleted *wt* recipients as described. Recipient mice were sacrificed 17 hours later, and quantities of recovered B220^+^ donor cells in the spleen were determined by FACS analysis.

## References

[1] Lanier LL (2008). Up on the tightrope: Natural killer cell activation and inhibition.. Nat Immunol.

[2] Yokoyama WM, Altfeld M, Hsu KC (2010). Natural killer cells: Tolerance to self and innate immunity to viral infection and malignancy.. Biol Blood Marrow Transplant.

[3] Karre K, Ljunggren HG, Piontek G, Kiessling R (1986). Selective rejection of H-2-deficient lymphoma variants suggests alternative immune defence strategy.. Nature.

[4] Kim S, Poursine-Laurent J, Truscott SM, Lybarger L, Song YJ (2005). Licensing of natural killer cells by host major histocompatibility complex class I molecules.. Nature.

[5] Fernandez NC, Treiner E, Vance RE, Jamieson AM, Lemieux S (2005). A subset of natural killer cells achieves self-tolerance without expressing inhibitory receptors specific for self-MHC molecules.. Blood.

[6] Hoglund P, Ohlen C, Carbone E, Franksson L, Ljunggren HG (1991). Recognition of beta 2-microglobulin-negative (beta 2m-) T-cell blasts by natural killer cells from normal but not from beta 2m- mice: Nonresponsiveness controlled by beta 2m- bone marrow in chimeric mice.. Proc Natl Acad Sci U S A.

[7] Liao NS, Bix M, Zijlstra M, Jaenisch R, Raulet D (1991). MHC class I deficiency: Susceptibility to natural killer (NK) cells and impaired NK activity.. Science.

[8] Dimasi N, Biassoni R (2005). Structural and functional aspects of the Ly49 natural killer cell receptors.. Immunol Cell Biol.

[9] Johansson S, Johansson M, Rosmaraki E, Vahlne G, Mehr R (2005). Natural killer cell education in mice with single or multiple major histocompatibility complex class I molecules.. J Exp Med.

[10] Brodin P, Karre K, Hoglund P (2009). NK cell education: Not an on-off switch but a tunable rheostat.. Trends Immunol.

[11] Joncker NT, Fernandez NC, Treiner E, Vivier E, Raulet DH (2009). NK cell responsiveness is tuned commensurate with the number of inhibitory receptors for self-MHC class I: The rheostat model.. J Immunol.

[12] Brodin P, Lakshmikanth T, Johansson S, Karre K, Hoglund P (2009). The strength of inhibitory input during education quantitatively tunes the functional responsiveness of individual natural killer cells.. Blood.

[13] Ljunggren HG, Van Kaer L, Ploegh HL, Tonegawa S (1994). Altered natural killer cell repertoire in tap-1 mutant mice.. Proc Natl Acad Sci U S A.

[14] Garbi N, Tan P, Diehl AD, Chambers BJ, Ljunggren HG (2000). Impaired immune responses and altered peptide repertoire in tapasin-deficient mice.. Nat Immunol.

[15] Bix M, Liao NS, Zijlstra M, Loring J, Jaenisch R (1991). Rejection of class I MHC-deficient haemopoietic cells by irradiated MHC-matched mice.. Nature.

[16] Fehling HJ, Swat W, Laplace C, Kuhn R, Rajewsky K (1994). MHC class I expression in mice lacking the proteasome subunit LMP-7.. Science.

[17] Caudill CM, Jayarapu K, Elenich L, Monaco JJ, Colbert RA (2006). T cells lacking immunoproteasome subunits MECL-1 and LMP7 hyperproliferate in response to polyclonal mitogens.. J Immunol.

[18] Zaiss DM, de Graaf N, Sijts AJ (2008). The proteasome immunosubunit multicatalytic endopeptidase complex-like 1 is a T-cell-intrinsic factor influencing homeostatic expansion.. Infect Immun.

[19] de Graaf N, van Helden MJ, Textoris-Taube K, Chiba T, Topham DJ (2011). PA28 and the proteasome immunosubunits play a central and independent role in the production of MHC class I-binding peptides in vivo.. Eur J Immunol.

[20] Chen W, Norbury CC, Cho Y, Yewdell JW, Bennink JR (2001). Immunoproteasomes shape immunodominance hierarchies of antiviral CD8(+) T cells at the levels of T cell repertoire and presentation of viral antigens.. J Exp Med.

[21] Pang KC, Sanders MT, Monaco JJ, Doherty PC, Turner SJ (2006). Immunoproteasome subunit deficiencies impact differentially on two immunodominant influenza virus-specific CD8+ T cell responses.. J Immunol.

[22] Moebius J, van den Broek M, Groettrup M, Basler M (2010). Immunoproteasomes are essential for survival and expansion of T cells in virus-infected mice.. Eur J Immunol.

[23] Hayakawa Y, Smyth MJ (2006). CD27 dissects mature NK cells into two subsets with distinct responsiveness and migratory capacity.. J Immunol.

[24] Chiossone L, Chaix J, Fuseri N, Roth C, Vivier E (2009). Maturation of mouse NK cells is a 4-stage developmental program.. Blood.

[25] Lucas M, Schachterle W, Oberle K, Aichele P, Diefenbach A (2007). Dendritic cells prime natural killer cells by trans-presenting interleukin 15.. Immunity.

[26] Jonsson AH, Yang L, Kim S, Taffner SM, Yokoyama WM (2010). Effects of MHC class I alleles on licensing of Ly49A+ NK cells.. J Immunol.

[27] Brodin P, Lakshmikanth T, Mehr R, Johansson MH, Duru AD (2010). Natural killer cell tolerance persists despite significant reduction of self MHC class I on normal target cells in mice.. PLoS One.

[28] Sun JC, Lanier LL (2008). Cutting edge: Viral infection breaks NK cell tolerance to “missing self”.. J Immunol.

[29] Polakos NK, Klein I, Richter MV, Zaiss DM, Giannandrea M (2007). Early intrahepatic accumulation of CD8+ T cells provides a source of effectors for nonhepatic immune responses.. J Immunol.

